# Metalloproteinase 14 (MMP-14) and hsa-miR-410-3p expression in human inflamed dental pulp and odontoblasts

**DOI:** 10.1007/s00418-019-01811-6

**Published:** 2019-09-05

**Authors:** Aniela Brodzikowska, Agata Gondek, Beata Rak, Wiktor Paskal, Kacper Pełka, Agnieszka Cudnoch-Jędrzejewska, Paweł Włodarski

**Affiliations:** 1 The Department of Conservative Dentistry, Medical University of Warsaw, Miodowa 18, 00-246 Warsaw, Poland; 2 Laboratory of Centre for Preclinical Research, Department of Methodology, Medical University of Warsaw, Banacha 1b, 02-097 Warsaw, Poland; 3 Laboratory of Centre for Preclinical Research, Department of Experimental and Clinical Physiology, Medical University of Warsaw, Banacha 1b, 02-097 Warsaw, Poland; 4 Postgraduate School of Molecular Medicine, Medical University of Warsaw, Warsaw, Poland; 5 Department of Internal Medicine and Endocrinology, Medical University of Warsaw, Banacha 1a, Warsaw, Poland

**Keywords:** MMP-14, microRNA-410, Tooth pulp, Odontoblasts

## Abstract

The objective of this study is to evaluate MMP-14 expression in odontoblasts and in the bulk of dental pulp of teeth with pulpitis; to determine the expression of microRNA-410 (miR-410) in pulp tissue, since sequence analysis suggests that miR-410 has potential binding site on MMP-14’s 3′UTR, and hence, can regulate expression of the latter one. Tissue samples of dental pulp from teeth with pulpitis and healthy (control) were formalin fixed and paraffin embedded (FFPE). Samples were examined using immunohistochemical staining for MMP-14 and the expression of miR-410 was evaluated using qRT-PCR. In both, healthy and inflamed pulp odontoblasts stained more intensively than remaining pulp tissue, but this difference was not statistically significant. More positive staining was observed in inflamed pulps compared to healthy pulps. Expression of miR-410 was found significantly lower in inflamed pulps than in healthy ones. In the two examined zones, odontoblasts and remaining pulp, miR-410 was expressed on a similar level. No statistically significant correlation of miR-410 and MMP-14 expression was found. We showed that inflammation changes the MMP-14 expression in pulp tissue and odontoblasts. This study demonstrates for the first time miR-410 expression in human dental pulp and that expression of this microRNA was downregulated in inflamed dental pulp and odontoblasts.

## Introduction

Pulpal inflammation is caused by microbes, and physical or chemical irritants. The pulp injury causes cellular damage and release of nonspecific proinflammatory mediators, e.g., histamine, bradykinin, neurokinins, and prostaglandins (Cooper et al. 2010). These factors cause vasodilatation, increased blood flow, exudate formation, and edema. The pulpal inflammation usually progresses slowly. Blood stasis leads to increased red blood cell aggregation, blood viscosity, and CO_2_ levels, as well as decreased pH levels and waste product removal. Carious enamel and dentin contain numerous bacteria capable of eliciting inflammatory reactions in pulp. These reactions are not often caused by direct exposure to bacteria, but instead to their toxins that penetrate via dentinal tubules into the pulp. The bacterial antigens and lipopolysaccharides (LPS) increase the levels of immunoglobulins, prostaglandins, and other proinflammatory mediators in the infected pulp (Nakanishi et al. 1995). Bacterial components and inflammatory factors stimulate the neutrophil degranulation and secretion by monocytes/macrophages (Chang et al. 2001; Lu et al. 2002). The released IL-1 and tumor necrosis factor-α (TNF-α) are able to induce MMP-1, MMP-2, and the expression of tissue inhibitor for metalloproteinases-1 (TIMP-1) gene in pulpal cells (Chang et al. 2001; Lin et al. 2001). Stimulation by black-pigmented Bacteroides elevates MMP-2 production (Chang et al. 2002) and anaerobic bacterial extracts provoke pulp cells to excrete MMP-1 and MMP-2 as well as TIMP-1 (Nakata et al. 2000). The levels of MMP-1, -2, and -3 are significantly higher in acute pulpitis than in normal pulp tissue (Shin et al. 2002).

The most common cause of pulp inflammation and necrosis is caries. More than 460 bacterial taxa have been identified in endodontic infections (Siqueira and Rocas 2009). The tooth pulp is composed of connective tissue that contains numerous nerve fibers, vessels, and undifferentiated mesenchymal cells. The connective tissue core is surrounded by a layer of odontoblasts with their processes (Tomes’ fibers) penetrating to dentinal tubules. Therefore, caries-induced inflammation initially affects odontoblasts—the outermost cells in the pulp (Love and Jenkinson 2002). It has been shown that the enzymatic degradation of the extracellular matrix (ECM) plays an important role in the development of inflammation (Gusman et al. 2002).

Matrix metalloproteinases (MMPs) form a group of proteases that belong to a family of structurally related zinc-dependent proteolytic enzymes known to play a key role in the catabolic turnover of extracellular matrix (ECM) and basement membrane (MB) components. MMPs are divided according to their substrate specificities and structures to interstitial collagenases, gelatinases, membrane-type MMPs, stromelysins, matrilysins, and other MMPs. MMPs regulate the activity of several non-ECM bioactive substrates including growth factors, cytokines, chemokines, and cell receptors with determining the tissue microenvironment. MMPs play a significant role in embryonal development, wound healing, and cell differentiation during tissue remodeling. The overexpression of several MMPs is involved in tumor invasion, metastasis, inflammation, and even cell destruction (Manicone and McGuire 2008). MMPs have recently been found also in pulpal tissue and odontoblasts, where they play a role in dentin matrix formation and modulation during caries progression and secondary dentin formation (Tjaderhane et al. 2001).

Cysteine cathepsin found in dentin has the ability to activate latent MMPs. The cathepsin activity increases with increasing depth of lesion. In carious lesions, cathepsin tends to influence the process through activation of latent MMPs. Most recent studies have indicated role of cysteine cathepsins in dentine caries by degradation of collagen through activating MMPs (Tjaderhane et al. 2013; Vidal et al. 2014).

In inflamed tissues, MMPs are synthesized both by structural cells (e.g., fibroblasts, keratinocytes, mast cells, osteoblasts, and odontoblasts), and by inflammatory cells (monocytes, macrophages, lymphocytes T, and neutrophils) (Chaussain-Miller et al. 2006; Gusman et al. 2002). In pathological conditions (inflammation, cancer, and degenerative diseases), increased activity of MMPs is not effectively inhibited by the tissue inhibitors of metalloproteinases (TIMPs). The result of this imbalance is the partial degradation of ECM and tissue injury (Baker et al. 2002).

Palosaari et al. have demonstrated the expression of following metalloproteinases in normal, mature human dental pulp: MMP-1, -2, -9, -10, -11, -13, -14, -15, -16, -17, -19, -20, and -23. Interestingly, MMP-7, -8, -24, and -25 were present exclusively in odontoblasts. It has been shown that expression of MMP -2, -10, -11, -14, and -20 was at least five times greater in odontoblasts than in remaining dental pulp cells (Palosaari et al. 2003). The authors suggested the potential role of MMP-14 in the activation of the other odontoblasts-derived MMPs. The expression of MMP-14 on the cell surface may initiate a cascade of proteases and the degradation of the ECM. It has also been demonstrated that the expression of MMP-14 in caries was increased in odontoblasts on the mRNA and protein level. However, the expression of MMP-14 was 13 times greater in odontoblasts than in the rest of the pulp (Palosaari et al. 2003). In pulpitis, an increased amount of MMP-2, -9 (Accorsi-Mendonca et al. 2013; Zehnder et al. 2011), MMP-8 (Wahlgren et al. 2002), and MMP-13 (Evrosimovska et al. 2012) was observed.

Metalloproteinase 14 (MMP-14), also called a membrane-type 1 metalloproteinase (MT1-MMP), is one of the metalloproteinases, which may play a critical role in healthy and inflamed pulp. Its structure contains a transmembrane domain, which passes through the cell membrane, and a short cytoplasmic C-terminal domain (Sternlicht and Werb 2001). MMP-14 directly cleaves components of the extracellular matrix, including fibronectin, collagen, and gelatin (Pei and Weiss 1996). It also activates pro-MMP-2 (Hernandez-Barrantes et al. 2000; Strongin et al. 1995) and takes part in the cell invasion to ECM (Nakahara et al. 1997). MMP-14 allows the cell migration, and like other metalloproteinases, plays an important role in tumor invasion and metastasis (Itoh and Seiki 2006; Sabeh et al. 2004). MMP-14 also can modulate inflammatory response of macrophages (Shimizu-Hirota et al. 2012). However, exact function of MMP-14 and other MMPs in the pulp and odontoblasts is unknown. The phenotype of MT1-MMP knockout mice partially explains the role of metalloproteinases in the development and growth of bones and teeth (Beertsen et al. 2002, 2003). The lack of such MMPs leads to reduction of the teeth growth and inhibition of bone formation (Beertsen et al. 2002, 2003).

MicroRNAs (miRNAs) are small non-coding RNA molecules, which bind to the 3′-untranslated region (UTR) of target messenger RNAs and consequently cause degradation of this mRNA or inhibit its translation (Ambros 2004). In result, miRNAs negatively regulate expression of targeted genes. MicroRNAs are involved in regulation of inflammation and immune response to bacterial infection of the dental pulp (Sonkoly et al. 2008). Modulated expression of miRNAs in the inflamed pulp of the tooth indicates that these molecules are involved in the inflammatory response in diseased dental pulp (Hui et al. 2017; Kong et al. 2014; Zhong et al. 2012, 2017). As previously shown, MMP-14 is potential target for hsa-miR-410-3p (miR-410) (Rak et al. 2016).

The aim of the study was to evaluate MMP-14 expression in odontoblasts and in the bulk of dental pulp of teeth with pulpitis. We analyzed also the expression of miR-410 in both zones of the tissue, since sequence analysis suggests that miR-410 has potential binding site on MMP-14’s 3′UTR, and, hence, can regulate expression of the latter one (Rak et al. 2016).

## Materials and methods

18 samples of dental pulp have been removed from the teeth extracted for orthodontic indications or have been extirpated from teeth with pulpits. Tissue samples were then formalin fixed and paraffin embedded (FFPE). Histopathological evaluation of HE-stained sections verified the correctness of clinical classification as an inflamed or healthy (control) pulp. Next, samples were examined using immunohistochemical staining and qRT-PCR. All experiments were performed in the Laboratory of Center for Preclinical Research of the Department of Methodology, Medical University of Warsaw.

### Immunohistochemistry

Immunohistochemical staining (IHC) was performed on 10 µm sections from FFPE tissues. Sections were deparaffinized according to standard protocol and antigens were retrieved in citrate buffer. The activity of endogenous peroxidase was diminished by incubation with 3% solution of hydrogen peroxide in methanol. To block nonspecific antibody binding, the sections were incubated with 2.5% normal horse serum for 40 min. Then, sections were incubated overnight in a humid chamber with primary polyclonal anti-MMP-14 antibody (cat no ab3644, Abcam, Cambridge, UK) in concentration 5 µg/ml at 4 °C. At the same time, negative controls were incubated with blocking solution instead of the primary antibody. Incubation with peroxidase-conjugated secondary antibody (ImmPRESS™, catalog no MP-7401, Vector Laboratories, CA, USA) was carried out for 40 min at the room temperature. Reaction products were visualized by DAB chromogen (Dako Liquid DAB + Substrate Chromogen System, Dako, Denmark) and hematoxylin staining. The results were evaluated using microscope with scanning feature (PALM Robo, Carl Zeiss AG, Oberkochen, Germany). Sections were scanned with 20× objectives; images were stitched and processed with GIMP (The GIMP team, GIMP 2.8.10). The expression of MMP-14 was scored and quantified according to stain intensity using IHC Profiler in ImageJ (ImageJ v 1.48).

### Laser capture microdissection

Laser capture microdissection (LCM) was performed on three healthy and three inflamed dental pulp tissue samples. Before the dissection, 10 µm sections were stained with hematoxylin and eosin without mounting the cover glass. From each sample, 15 mm^2^ of odontoblasts or pulp tissue without odontoblasts were dissected into separate tubes (AdhesiveCap, Carl Zeiss AG, Oberkochen, Germany). RoboLPC mode was used for LCM (PALM Robo, Carl Zeiss AG, Oberkochen, Germany).

### MicroRNA isolation and qRT-PCR

Total RNA was isolated from the excised tissues using RecoverALL Total Nucleic Acid Isolation Kit (Ambion™, Life Technologies, USA). Reverse transcription was performed with TaqMan miRNA primers and TaqMan microRNA Reverse Transcription Kit (Applied Biosystems, Life Technologies Corporation, USA). Expression of miR-410 was determined by real-time PCR using Taqman MicroRNA Assay (Assay ID 001274; miRBase Accession Number MI0002465; Stem-loop Sequence GGUACCUGAGAAGAGGUU-GUCUGUGAUGAGUUCGCUUUUAUUAAUGACGAAUAUAACACAGAUGGCCUGUUUUCAGUACC; Applied Biosystems, Life Technologies Corporation, USA), sensiFAST Probe Lo-ROX Mix (Bioline, USA) with the Applied Biosystems 7500 Fast Real-Time PCR System, and 7500 Software V2.0.6 (Life Technologies Corporation 2011). U6 snRNA was used as an endogenous control (Assay ID 001973; NCBI Accession # NR_004394; Control Sequence: GTGCTCGCTTCGGCAGCACATATACTAAAATTGGAACGATA-CAGAGAAGATTAGCATGGCCCCTGCGCAAGGATGACACGCAAATTCGTGAAGCGTTCCATATTTT). PCR conditions were in accordance with the manufacturer’s protocol. The relative expression of miR-410 was calculated automatically by the comparative Ct method.

### Statistics

Analysis of IHC results and relative Ct from qRT-PCR were calculated with Excel 2016 (Microsoft Corporation, Redmond, WA, USA). Plots and statistical analysis of mir-410 expression and anti-MMP 14 staining including Wilcoxon signed-rank, *U* Mann–Whitney, R Spearman’s rank correlation, and one-way ANOVA tests were performed with Statistica 11 (Statsoft Inc., Dell Statistica, Tulsa, OK, USA) and the GraphPad Prism statistics software (GraphPad Prism 6, San Diego, CA, USA). The *p* value of < 0.05 was considered statistically significant.

## Results

### MMP-14 expression in healthy and inflamed dental pulp

**Fig. 1:**
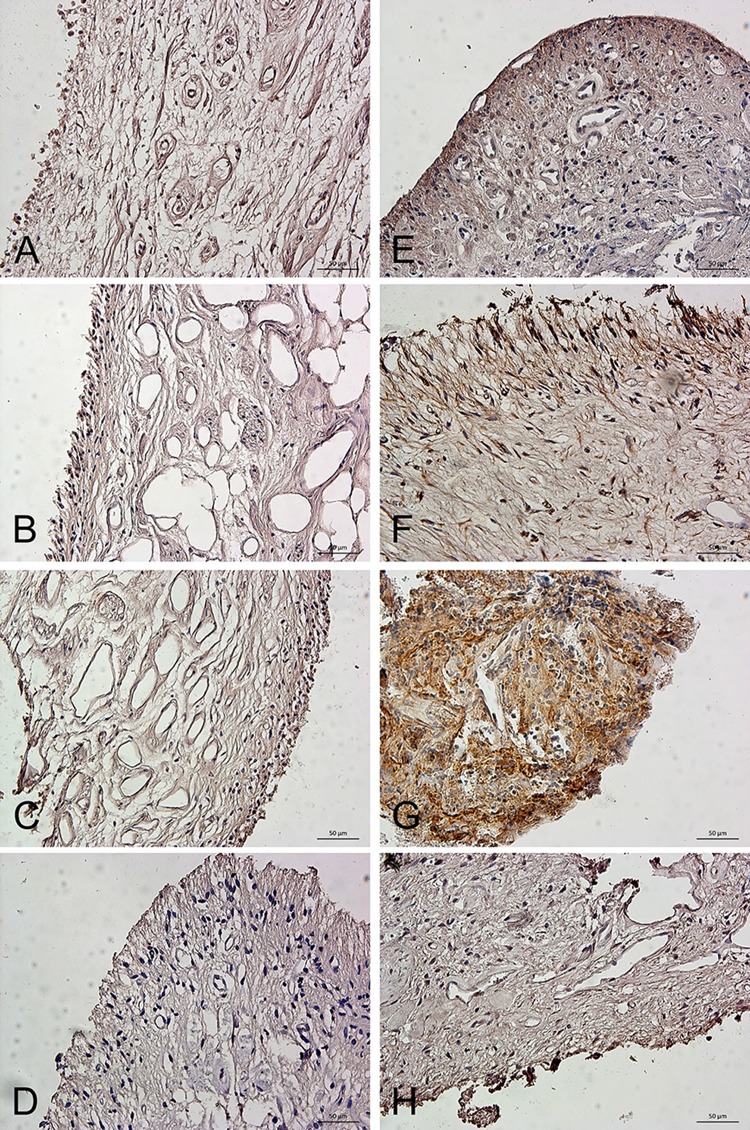
Healthy (**a**–**d**) and inflamed (**e**–**h**) dental pulp. Each specimen was obtained from distinct patient. Immunohistochemical staining for MMP-14. Scale bar = 5 µm

**Fig. 2:**
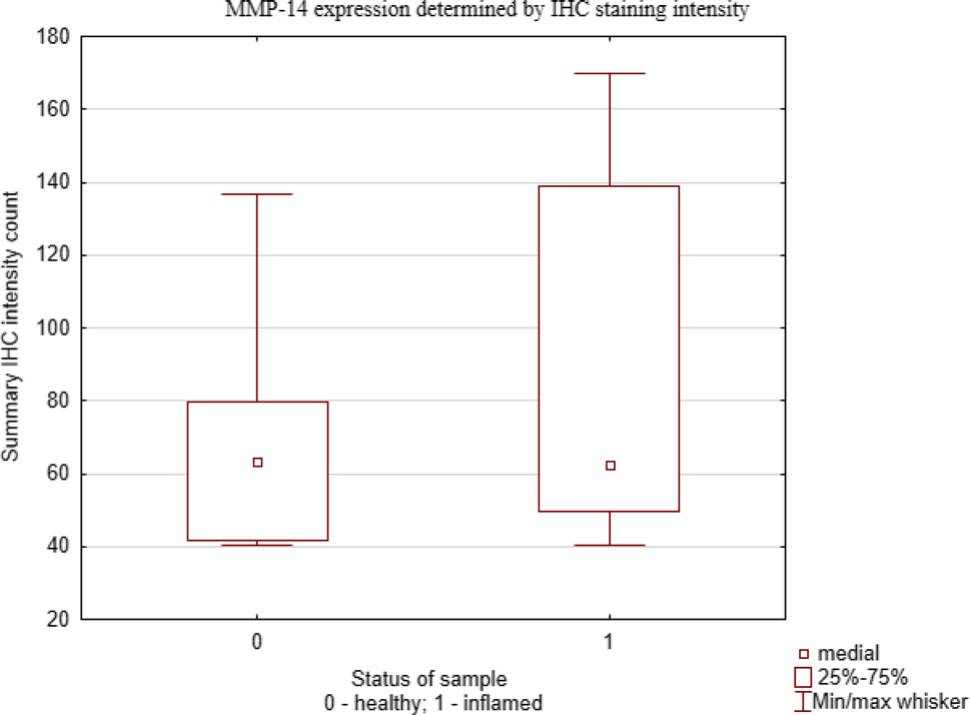
The quantitative analysis of MMP-14 expression determined by IHC staining intensity in healthy and in inflamed pulp tissue by IHC Profiler in ImageJ (ImageJ v 1.48)

The assessment of expression of MMP-14 was performed on 12 tissue samples by immunohistochemical staining. In both, healthy and inflamed pulp odontoblasts stained more intensively than remaining pulp tissue, but this difference was statistically insignificant (*p *= 0.071, Wilcoxon signed-rank test). More positive staining was observed in inflamed pulps compared to healthy pulps (Fig. 1). The quantitative analysis of the MMP-14 staining intensity of healthy and inflamed pulps is presented in Fig. 2. Although the expression of MMP-14 was higher in inflamed pulps, these differences are statistically insignificant (*U* Mann–Whitney and ANOVA tests).

### miR-410 expression in odontoblasts and dental pulp tissue

**Fig. 3:**
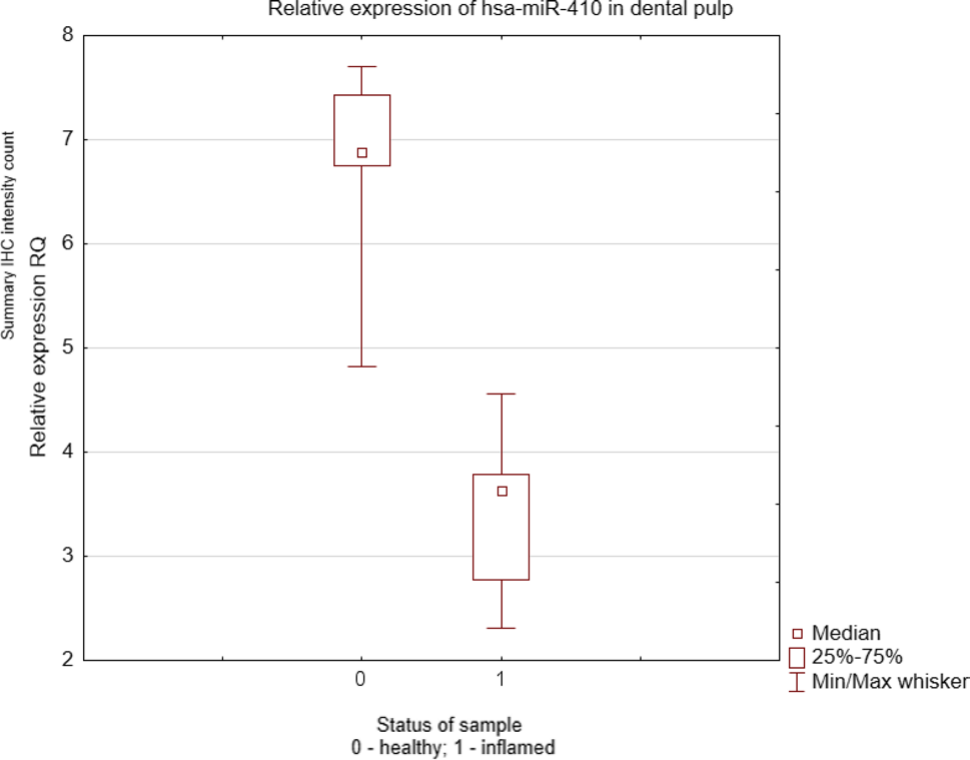
miR-410 expression level in healthy and inflamed pulp tissue. The expression of miR-410 was determined by qRT-PCR with U6 as an endogenous control and calculated by the comparative CT method

**Fig. 4:**
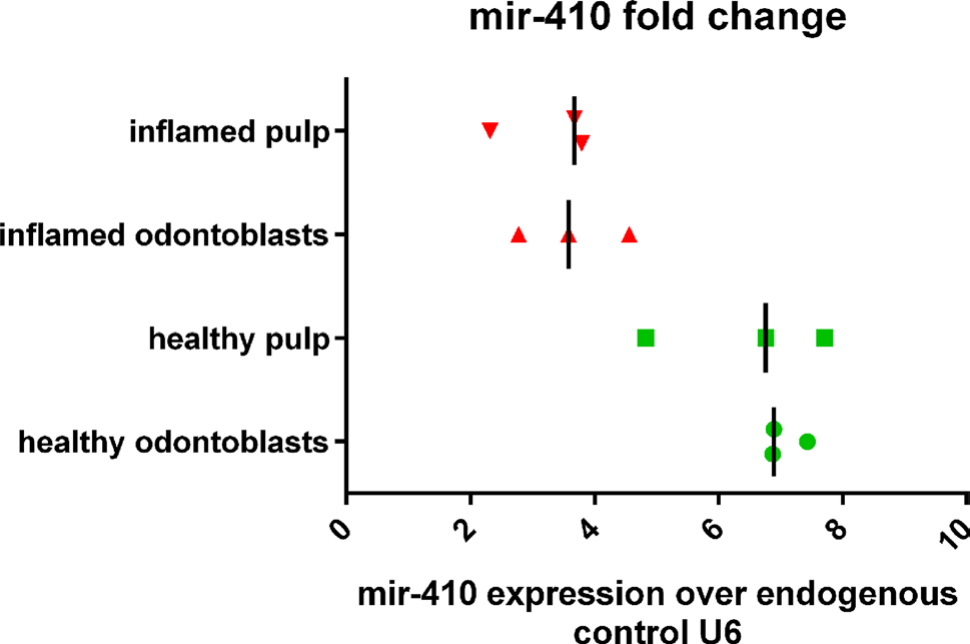
The relative level of the miR-410 expression over the endogenous control U6. The graph shows distinction into different subgroups: healthy, inflamed, pulp and odontoblasts. Median value is signed with line. The total level of the miR-410 expression in inflamed tissue was lower compared to healthy ones

The miR-410 expression in healthy and inflamed pulp tissue is presented in Fig. 3. Expression of this microRNA was significantly lower in inflamed pulps than in healthy ones *p *= 0.0021 and *p *= 0.0025 (Mann–Whitney *U* and one-way ANOVA test). The analysis of the miR-410 expression both in odontoblasts and pulp tissue is shown in Fig. 4. In the two examined zones, odontoblasts and remaining pulp, miR-410 was expressed on a similar level *p *> 0.05 (Wilcoxon signed-rank test). However, no statistically significant correlation of miR-410 and MMP-14 expression was found (*r *= − 0.028, *p *> 0.05, *R* Spearman’s rank correlation test) neither in healthy nor in inflamed pulp.

## Discussion

The inflammation of the pulp usually results when bacterial toxins pass through dentin and reach the pulp (Birkedal-Hansen 1995). The increased levels of MMPs in inflamed pulp suggest that MMPs play an important role in the extracellular tissue degradation and in the disease progression. On the other hand, MMPs are important mediators during physiological tissue remodeling (Jain and Bahuguna 2015), being a key component of the local protective mechanisms of the dentin–pulp complex in cases of inflammation. Most likely MMPs play a role during the inflammation of the pulp by mediating in the disintegration of the pulp connective tissue, which enables odontoblast-like cells to migrate and form reparative dentin (Evrosimovska et al. 2012).

The understanding of the role of MMP activity in the oral environment is incomplete without discussing the formation of human caries lesions. In dental caries, demineralization is caused by microbial acids, and degradation of dentinal organic matrix was thought to be carried out solely by microbial proteolytic enzymes responsible for the degradation of dentine organic matrix. Presence of both pre- and active forms of MMP-8, MMP-2, and MMP-9 in human dental carious lesions suggests; however, their active role in the process. MMP-8, MMP-2, MMP-3, MMP-9, MMP-14, and MMP-20 are the main MMPs identified in pulp, odontoblasts, predentine, and dentine (Sulkala et al. 2004, 2007). MMP activity has been found to decrease with age in both active and chronic carious lesions (Nascimento et al. 2011).

Salivary MMPs also tend to have a significant contribution towards dentine matrix degradation during the carious process. Endogenous MMP-2 contained in sound dentine is activated during the carious process. Caries also tends to increase the level of endogenous MMP-2 synthesis. Acidic pH of carious dentin may both induce MMP production by the odontoblasts as well as their activation, thereby potentiating MMP proteolytic capacity, leading to enhanced dentine matrix degradation (Toledano et al. 2010).

Sorsa et al. demonstrated the contribution of both pro- and active forms of MMP-8, -2, and -9. After the demineralization of dentin by bacterial acids, enzymes contained in plaque and saliva and produced by odontoblasts break down the dentine matrix, consisting of collagen type I and small amounts of collagen type V. Non-collagenous proteins, including dentine matrix protein I, osteopontin, and dentine sialoprotein, have also been suggested as activators of MMP pro-forms taking part in dentin matrix degradation (Sorsa et al. 2004).

MMP-14 cleaves the collagen type I, II, and III, fibronectin, laminin 1 and 5, vitronectin, and proteoglycan (Koshikawa et al. 2004; Ohuchi et al. 1997). By contrast, it does not degrade type IV collagen, the main component of the basement membrane, but activates pro-MMP-2, which is capable of doing this (Okada et al. 1990).

In this study, the expression of MMP-14 in the pulp was evaluated by immunohistochemical staining. MMP-14 expression was found to be higher in inflamed pulp than healthy one. Also odontoblasts layer stained more intensively than other pulp tissue cells. These results, however, were not statistical significant, which may be due to the relative small number of samples. The presence of MMP-14 was demonstrated in mature human dental pulp and odontoblasts by Palosaari et al. (2002). These authors suggested the potential role of MMP-14 in the activation of the other odontoblast-derived MMPs. The expression of MMP-14 on the cell surface may initiate a cascade of proteases and degradation of ECM. It has also been demonstrated that in caries, the expression of MMP-14 was increased in odontoblasts on mRNA and protein level (Charadram et al. 2012). MMP-14 was upregulated during the formation of reactionary dentin.

MMP-14, besides pro-MMP-2 (Lehti et al. 1998), also activates pro-MMP-13 (Cowell et al. 1998; Knauper et al. 1996) and pro-MMP-20 (Palosaari et al. 2003). For example, MMP-13, also known as collagenase-3, degrades various components of the basement membrane and is highly effective in digestion of collagen type II (Jain and Bahuguna 2015). It was found that MMP-13 in rat pulp injury model plays critical roles in angiogenesis and pulp wound healing (Zheng et al. 2009). Whereas the study of Sulkala et al. (2002) demonstrates that in the external irritation, MMP-20 in dentin fluid originates from odontoblast and is secreted in tubular dentine. Therefore, MMP-20 may be involved in the defense responses; for example, the formation of secondary dentine, but may also be a component of the normal dentinal fluid. In addition, Giannelli et al. (1997) demonstrated that MMP-2 cleaves laminin 5 and plays a crucial role in cell migration during tissue remodeling and tumor invasion. Thus modifications of extracellular matrix components are necessary for cell migration. It has also been suggested that MMP-14 is essential for cell migration over laminin 5, while MMP-2, activated by MMP-14, plays an auxiliary role and may amplifying MMP-14 effects (Koshikawa et al. 2000, 2004).

All of the data discussed above show that various proteases are engaged in pulpitis, nonetheless, our present study was limited to examine solely the expression of MMP-14. Many studies point to altered expression of miR-410 in a variety of tumors (Guo et al. 2015; Li et al. 2015; Palumbo et al. 2016; Shen et al. 2014; Wang et al. 2014, 2016; Zhang et al. 2016), which proves its participation in their development as well as in tumor metastasis. Inflammation also affects the expression of microRNA. Zhong et al. (2012) determined the expression of miRNAs in healthy and inflamed human dental pulps and found that 33 miRNAs were downregulated in inflammatory pulps. In our study, mir-410 was also downregulated in the inflamed pulps. According to other study on miR-410, this molecule acts as an inflammatory suppressor via NF-κB signaling pathway and HMGB-1 (Wang et al. 2019; Xiong et al. 2017). Recently, examined intranasal administration of synthetic mir-410 led to reduced inflammation in asthmatic mice through IL4 and IL-13 depletion (Jin et al. 2019). These studies not only explain observed downregulation in inflamed dental pulp, but also increase the importance of explaining the role of mir410 in dental pulpitis as a potential, direct anti-inflammatory target. However, there was not any significant correlation between expression of MMP-14 and miR-410 in human dental pulp. We are aware that this finding may be the result of the small number of studied samples in each group. In current report, we confirmed overexpression of MMP-14 on 12 samples, while mir-410 was studied on six tissue samples (three healthy and three inflamed with the distinction to pulp and odontoblasts). Results presented in this report are in accordance with our previous findings in endometrial cancer (Rak et al. 2016).

To the best of our knowledge, this is the first study that demonstrates miR-410 expression in human dental pulp. We have shown here the expression pattern of MMP-14 in human dental pulp and odontoblasts in both: healthy and inflamed pulp and illustrated how inflammation changes the MMP-14 expression in pulp tissue including odontoblasts. MMP-14, due to its ability to activate another pro-MMPs, may have a crucial role during extracellular matrix remodeling an inflammation. The expression of miR-410 was downregulated in inflamed dental pulp and odontoblasts. These findings confirmed the intricate and specific role of miR-410 as an inflammation inhibitor and suggest that this microRNA must also play a role in pulpitis pathogenesis. Further research is needed to elucidate the targets by which miR-410 can potentially modulate the host response in pulpal disease.
